# Systemic Chemotherapy for Advanced Hepatocellular Carcinoma: Past, Present, and Future

**DOI:** 10.3390/diseases3040360

**Published:** 2015-12-01

**Authors:** Masafumi Ikeda, Shuichi Mitsunaga, Izumi Ohno, Yusuke Hashimoto, Hideaki Takahashi, Kazuo Watanabe, Kumiko Umemoto, Takuji Okusaka

**Affiliations:** 1 Department of Hepatobiliary and Pancreatic Oncology, National Cancer Center Hospital East, Kashiwa 277-8577, Japan; E-Mails: smitsuna@east.ncc.go.jp (S.M.); ioono@east.ncc.go.jp (I.O.); yusuke.h914@gmail.com (Y.H.); hidetaka@east.ncc.go.jp (H.T.); kazuowat@east.ncc.go.jp (K.W.); kumemoto@east.ncc.go.jp (K.U.); 2 Department of Hepatobiliary and Pancreatic Oncology, National Cancer Center Hospital, Tokyo 104-0045, Japan; E-Mail: tokusaka@ncc.go.jp

**Keywords:** hepatocellular carcinoma, chemotherapy, sorafenib, immune-oncologic agents, individualized treatment

## Abstract

Systemic chemotherapy is one of the most important treatment modalities for advanced hepatocellular carcinoma (HCC). Before the introduction of sorafenib, cytotoxic agents, hormonal therapies, or many combinations of these were the mainly used modalities for systemic chemotherapy of advanced HCC. However, such regimens were of only limited value in clinical practice, because some randomized controlled studies comparing promising regimens with no treatment or doxorubicin alone failed to show any overall survival advantage. In two pivotal phase III placebo-controlled studies, the SHARP trial and the Asia-Pacific trial, sorafenib was demonstrated to significantly delay the time to progression and the overall survival time in patients with advanced HCC. Therefore, sorafenib therapy has come to be acknowledged as a standard therapy for advanced HCC worldwide. After the introduction of sorafenib, a number of phase III trials of various molecular-targeted agents *vs.* sorafenib as first-line chemotherapy and of various molecular-targeted agents *vs.* placebo as second-line chemotherapy have been conducted to determine if any of these agents could offer a survival benefit, however, none of the agents examined so far has been demonstrated to provide any survival benefit over sorafenib or placebo. Recently, favorable treatment efficacies have been reported in some clinical trials of molecular-targeted agents in the biomarker-enriched population. Development of individualized cancer treatments using molecular-targeted agents based on the results of genome-sequencing is aggressively ongoing. Furthermore, immune-oncologic agents, such as anti-CTLA-4 antibody and anti-PD-1/PD-L1 antibody, have been reported to provide promising outcomes. Thus, various novel systemic chemotherapeutic agents are currently under development, and further improvements in the treatment outcomes are expected.

## 1. Introduction

Hepatocellular carcinoma (HCC) is the sixth most common of all malignancies and third most common cause of cancer-related death in the world [1,2], while ranking fifth among the causes of death from cancer in Japan [3]. Its incidence continues to increase worldwide, while the number of deaths from HCC has been gradually decreasing in Japan. The main reasons for this decreasing trend of death from liver cancer in Japan are considered to be the widespread screening for hepatitis B or C viral infection, which interrupts transmission of viral infection via transfusion and the establishment of treatments for hepatitis B or C viral infection [3]. Although a wide range of therapeutic options are available for HCC, the efficacy of these methods remains unsatisfactory and the prognosis of patients with HCC is still poor [2,3,4,5,6]. Curative therapies, such as hepatic resection and liver transplantation, are applicable to only a small proportion of patients with HCC because of poor liver function, tumor spread, or both. Local treatments, such as radiofrequency ablation or transarterial chemoembolization, have been reported to be useful for treating patients with unresectable HCC, but unfortunately, in most patients with HCC, the disease recurs/relapses, and progresses to an advanced stage for which effective local treatments are not yet available [2,3,4,5,6]. For patients with advanced stage disease, systemic chemotherapy is adopted as one of the most important treatment modalities. At present, among the systemic chemotherapy regimens, sorafenib is the standard of care for advanced HCC, because it has been demonstrated to significantly delay the time to progression and prolong the overall survival time in patients with advanced HCC in two pivotal phase III placebo-controlled studies [7,8]. This article reviews the past status, present status, and future direction of chemotherapy for advanced HCC: pre-sorafenib era, sorafenib era, and post-sorafenib era.

## 2. Past: Pre-Sorafenib Era

Before the introduction of sorafenib, cytotoxic agents, hormonal therapies, or combinations of these agents were main systemic chemotherapeutic modalities employed for the treatment of advanced HCC (Table 1). However, these are of only limited value in clinical practice. Various clinical trials conducted after the 1980s using different single agents reported overall response rates of 0%–20%. Combination chemotherapy with cytotoxic agents yielded higher response rates [9,10,11,12,13], however, some randomized controlled studies comparing promising combination therapies with no treatment or single agents failed to show any advantage in terms of the overall survival [14,15,16,17,18,19,20].

In 1988, a randomized controlled trial of doxorubicin *vs.* best supportive care was conducted in patients with advanced HCC [16]. In this study, doxorubicin showed significant survival benefit as compared to best supportive care in patients with advanced HCC (median: doxorubicin, 10.6 weeks; best supportive care, 7.5 weeks; *p*-value = 0.036). However, the incidence rates of serious adverse events such as sepsis and cardiac toxicities were very high (25%), therefore, the drug did not come to be regarded as a standard chemotherapy agent for advanced HCC. A randomized phase III trial comparing doxorubicin alone with a combination chemotherapy regimen consisting of cisplatin, interferon α-2b, doxorubicin, and 5-fluorouracil (PIAF regimen) was conducted [18], however, it revealed no significant difference in survival between the two treatment arms (median: PIAF, 8.67 months; doxorubicin, 6.83 months; *p*-value = 0.83). Another randomized phase III trial of doxorubicin *vs.* combined chemotherapy with 5-fluorouracil, leucovorin, and oxaliplatin (FOLFOX4) conducted in patients with advanced HCC revealed a significant difference in the progression-free survival (median: FOLFOX4, 2.93 months; doxorubicin, 1.77 months; hazard ratio, 0.62; *p* < 0.001), but no significant difference in the overall survival (median: FOLFOX4, 6.40 months; doxorubicin, 4.97 months; hazard ratio, 0.80; *p* = 0.07) [20]. Therefore, FOLFOX4 has also not been acknowledged as a standard chemotherapy for advanced HCC, even though follow-up studies of this phase III trial demonstrated better survival benefit (hazard ration, 0.74; *p* = 0.03) [21]. Patients with advanced HCC tend to experience more severe hepatic toxicities and myelosuppression than those with other malignancies, as they frequently have liver cirrhosis, which is usually associated with compromised hepatic function, leukocytopenia, and thrombocytopenia [1,2,3,6,13]. This may be one of the main reason why it is difficult to recognize any significant survival benefit of combination regimens from randomized controlled trials.

Numerous randomized controlled trials of hormonal therapies [22,23,24,25,26,27,28,29,30,31], such as tamoxifen or octreotide, and interferon therapies [32,33,34,35] *vs.* best supportive care or placebo have been conducted worldwide. While some have demonstrated prolongation of survival, others have shown no survival benefit in the treatment arm. Thus, conflicting results have been obtained from clinical trials in patients with advanced HCC. Furthermore, some meta-analyses reported that none of the treatments offered any significant survival benefit [36,37,38,39], therefore, none have been recommended as a systemic treatment option for patients with advanced HCC.

In summary, in the pre-sorafenib era, no standard therapy for advanced HCC had been established, because randomized controlled trials and meta-analyses failed to confirm any survival benefit of cytotoxic regimens, hormonal therapies, or interferon therapies in patients with advanced HCC.

**Table 1:** Results of main randomized controlled trials of systemic chemotherapy for advanced HCC: Pre sorafenib-era.

Regimen	No. of Patients	Response Rate (%)	Overall Survival		Authors	Reported Year	Reference No.
			Median	*p*-value			
**Randomized Controlled Trials of Cytotoxic Agents**							
Etoposide	22	18	ND				
Doxorubicin	28	28	ND	ND	Melia WM	1983	[14]
Mitoxantrone	34	0	14 w				
Cisplatin	35	6	14 w	ND	Falkson G	1987	[15]
Doxorubicin	60	3.3	10.6 w				
Best supportive care	46	ND	7.5 w	0.036	Lai CL	1988	[16]
Tegaful-uracil	28	17.8	12.13 m				
Best supportive care	20	ND	6.20 m	<0.01	Ishikawa T	2001	[17]
Cisplatin, interferon α-2b, doxorubicin, 5-FU (PIAF)	94	20.9	8.67 m				
Doxorubicin	94	10.5	6.83 m	0.83	Yeo W	2005	[18]
Nolatrexed	222	1.4	22.3 w				
Doxorubicin	222	4.0	32.3 w	0.0068	Gish RG	2007	[19]
5-FU, Oxaliplatin, Leukovorin (FOLFOX4)	184	8.15	6.40 m				
Doxorubicin	187	2.67	4.97 m	0.07	Qin S	2010	[20]
**Randomized Controlled Trials of Hormonal Therapies**							
Tamoxifen (40 mg/d)	240	ND	15 m				
Best supportive care	237	ND	16 m	0.54	CLIP group	1998	[22]
Tamoxifen (120 mg/d)	120	ND	2.2 m				
Tamoxifen (60 mg/d)	74	ND	2.1 m				
Placebo	130	ND	2.7 m	0.01	Chow PK	2002	[23]
Tamoxifen, leuprorelin, flutamide	192	ND	135.5d				
Tamoxifen	184	ND	176 d	0.21	GETCH	2004	[24]
Tamoxifen (20 mg/d)	210	ND	4.8 m				
Best supportive care	210	ND	4.0 m	0.25	Barbare JC	2005	[25]
Antiandrogen, placebo	60	1.6	3.9 m				
LHRH agonist, placebo	62	3.2	2.7 m				
Antiandrogen, LHRH agonist	62	1.6	3.6 m				
Placebo, placebo	60	0	5.8 m	0.19	Grimaldi C	1998	[26]
Octreotide	60	0	4.7 m				
Placebo	59	0	5.3 m	0.59	Becker G	2007	[27]
Octreotide+tamoxifen	56	5	3 m				
Tamoxifen	53	3.7	6 m	0.609	Verset G	2007	[28]
Octreotide	135	0	6.53 m				
Placebo	137	2.9	7.03 m	0.34	Barbare JC	2009	[29]
Megestrol	21	ND	18 m				
Best supportive care	24	ND	7 m	0.009	Villa E	2001	[30]
Megestrol	135	ND	1.88 m				
Placebo	69	ND	2.14 m	0.16	Chow PK	2011	[31]
**Randomized Controlled Trials of Interferon Therapies**							
Interferon α-2a	50	10	8.3 w				
Doxorubicin	25	0	4.8 w	NS	Lai CL	1989	[32]
Interferon α-2a	35	31.4	14.5 w				
Best supportive care	36	0	7.5 w	0.0471	Lai CL	1993	[33]
Interferon β	31	0	11.1 w				
Menogaril	34	0	23.1 w	NS	Falkson G	1995	[34]
Interferon α-2b	30	6.6	58% *				
Best supportive care	28	0	36% *	0.14	Llovet JM	2000	[35]

5-FU, 5-fluorouracil; GETCH, Groupe d'Etude et de Traitement du Carcinome Hépatocellulaire; * 1 year survival rate; ND, No data; NS, not significant.

## 3. Present: Sorafenib Era

Sorafenib is a multikinase inhibitor of Raf kinase, which is involved in cancer cell proliferation, and also of vascular endothelial growth factor receptor-2/-3 (VEGFR-2/-3) and platelet-derived growth factor receptor-beta (PDGFR-β), which are involved in peritumor neovascularization [40,41,42]. In two pivotal phase III placebo-controlled studies, the so-called SHARP trial [7] and the Asia-Pacific trial [8], sorafenib was demonstrated to significantly prolong the time to progression as well as the overall survival time in patients with advanced HCC. Therefore, sorafenib has come to be acknowledged as a standard therapy for advanced HCC, and is available worldwide. However, the efficacy is modest: the median survival is less than one year and the tumor response rate is less than 5%. Thus, there remains a critical and unmet need for aggressive development of newer and more effective agents for advanced HCC.

After the introduction of sorafenib, a number of phase III trials of various molecular-targeted agents *vs.* sorafenib as first-line treatment have been conducted to determine if any could offer a longer overall survival than sorafenib [43,44,45,46,47,48,49,50,51], however, none of the agents examined so far have been demonstrated to offer survival benefit over sorafenib. Furthermore, some phase III trials of various molecular-targeted agents *vs.* placebo have been conducted in HCC patients who were refractory or intolerant to sorafenib [52,53,54,55,56,57], to determine if any could offer a longer overall survival than placebo, however, none of the agents examined so far have been demonstrated to offer survival benefit over placebo.

## 4. Targeted Therapy: First-Line Chemotherapy

Various chemotherapeutic agents such as sunitinib, brivanib, linifanib, Sorafenib plus erlotinib, vandetanib, nintedanib, dovitinib, sorafenib plus doxorubicin, *etc.*, have been evaluated by randomized controlled trials worldwide as first-line treatment for patients with advanced HCC (Table 2).

### 4.1. Sunitinib

Sunitinib is an orally administered multitargeted tyrosine kinase inhibitor of VEGFR-1, VEGFR-2, VEGFR-3, PDGFR-α, PDGFR-β, and several other related tyrosine kinases, with antitumor and antiangiogenic activities. In a phase III trial of sunitinib *vs.* sorafenib [43], the overall survival in the sunitinib arm was not superior or equivalent, but significantly inferior to that in the sorafenib arm, although the progression-free survival did not differ significantly between the two treatment arms.

### 4.2. Brivanib

Brivanib is the first reported orally administered selective dual inhibitor of the FGF and VEGF receptor tyrosine kinases. In a phase III trial of brivanib *vs.* sorafenib [44], the predefined non-inferiority boundary for overall survival (non-inferiority margin, 1.08 of the upper limit of the 95% CI for the hazard ratio) was not met, although the overall survival, time to progression, objective response rate, and disease control rate were similar between the brivanib and sorafenib arms.

**Table 2:** Results of main randomized controlled trials worldwide as first-line and second-line treatment for patients with advanced HCC: Sorafenib-era.

Agents	*n*	RR (%)	DCR (%)	TTP/PFS (Median: Months)	Hazard Ratio (95% CI)	*p*-value	OS (Median: Months)	Hazard Ratio (95%CI)	*p*-value	Phase/Name of Trial	Authors Reported Year		Reference No.
**First Line Chemotherapy**													
Sunitinib	530	6.6%	50.8%	3.6	1.13 (0.99–1.30)	0.2286	7.9	1.30 (1.13–1.50)	0.0014	Phase III	Cheng AL		
Sorafenib	542	6.1%	51.5%	3.0	-		10.2			SUN1170	2013		[43]
Brivanib	577	12%	66%	4.2	1.01 (0.88–1.16)	0.8532	9.5	1.06 (0.93–1.22) *	0.3730	Phase III	Jofnson P		
Sorafenib	578	9%	65%	4.1	-		9.9	-		BRISK-FL	2013		[44]
Linifanib	514	13.0%	ND	5.4	0.759 (0.643–0.895)	0.001	9.1	1.046 (0.896–1.221)	ND	Phase III	Cainap C		
Sorafenib	521	6.9%	ND	4.0	-		9.8	-		LiGHT	2015		[45]
Sorafenib + Erlotinib	362	6.6%	43.9%	3.2	1.135 (0.944–1.366)	0.18	9.5	0.929 (0.781–1.106)	0.408	Phase III	Zhu AX		
Sorafenib + Placebo	358	3.9%	52.5%	4.0	-		8.5	-		SEARCH	2015		[46]
Vandetanib (10 mg)	25	0.0%	5.3%	1.70	0.64 (0.35–1.18)	0.15	5.75	0.44 (0.22–0.86)	0.02	Phase II			
Vandetanib (300 mg)	19	0.0%	16.0%	1.05	0.71 (0.38–1.36)	0.31	5.95	0.60 (0.30–1.19)	0.15		Hsu C		
Placebo	23	0.0%	8.7%	0.95	-		4.27	-			2012		[47]
Nintedanib	63	6.3%	68.3%	2.8	1.21 (0.73–2.01)	ND	10.2	0.94 (0.59–1.49)	ND	Phase II	Cheng AL		
Sorafenib	32	3.1%	84.4%	3.7			10.7	-			2015		[48]
Nintedanib	62	1.6%	82.3%	5.5	1.44 (0.81–2.57)	ND	11.9	0.88 (0.52–1.47)	ND	Phase II	Palmer D		
Sorafenib	31	6.5%	90.3%	4.6			11.4	-			2015		[49]
Dovitinib	82	6.1%	57.3%	4.1	1.42 (0.98–2.08)	ND	8.0	1.27 (0.90–1.79)	ND	Phase II	Cheng AL		
Sorafenib	83	10.8%	63.9%	4.1			8.5				2015		[50]
Sorafenib + Doxorubicin	47	4%	NA	6.4	0.5 (0.3–0.9)	0.02	13.7	0.49 (0.3–0.8)	0.006	Phase II	Abou-Alfa GK		
Doxorubicin	49	2%	NA	2.8	-		6.5	-			2010		[51]
**Second Line Chemotherapy**													
Brivanib	263	10%	61%	4.2	0.56 (0.42–0.76)	<0.001	9.4	0.89 (0.69–1.15) *	0.3307	Phase III	Llovet JM		
Placebo	132	2%	40%	2.7	-		8.2	-		BRISK-PS	2013	[52]	
Everolimus	362	2.2%	56.1%	3.0	0.93 (0.75–1.15)	ND	7.6	1.05 (0.86–1.27)	0.68	Phase III	Zhu AX		
Placebo	184	1.6%	45.1%	2.6			7.3	-		EVOLVE-1	2014	[53]	
S-1	222	5.4%	43.2%	2.6	0.60 (0.46–0.77)	<0.0001	11.1	0.86 (0.67–1.10)	0.2201	Phase III	Kudo M		
Placebo	111	0.9%	24.3%	1.4			11.2	-		S-CUBE	2015	[54]	
Axitinib	134	9.7%	31.1%	3.6	0.618 (0.438–0.871)	0.0	12.7	0.870 (0.620–1.222)	0.211	Phase III	Kang YK		
Placebo	68	2.9%	11.8%	1.9			9.7	-			2014	[55]	
GC33	121	ND	ND	2.6	0.98	0.93	6.8	0.99	0.97	Phase II	Yen CJ		
Placebo	60	ND	ND	1.5			6.7	-			2014	[56]	
Tigatuzumab (6/2 mg/kg) + Sorafenib	53	5.7%	54.8%	3.0	1.12 (0.69–1.80)	0.657	8.2	ND	0.303	Phase II			
Tigatuzumab (6/6 mg/kg) + Sorafenib	54	14.8%	68.5%	3.9	1.15 (0.73–1.81)	0.548	12.2	ND	0.659		Cheng AL		
Sorafenib	55	11%	54.6%	2.8	-		8.2	-			2015	[57]	

* 95.8% confidence interval; 6/2 mg/kg, 6 mg/kg loading, 2 mg/kg/week maintenance; 6/6 mg/kg, mg/kg loading, 6 mg/kg/week maintenance. RR, response rate; DCR, diasease control rate; TTP, time to progression; PFS, prgresion free survival; OS, overall survival; ND, no data.

### 4.3. Linifanib

Linifanib is a novel ATP-competitive inhibitor of all VEGF and PDGF receptor tyrosine kinases that lacks significant activity against representative cytosolic tyrosine kinases or serine/threonine kinases. In a phase III trial of linifanib *vs.* sorafenib [45], although a similar overall survival and a significantly favorable time to progression was observed in the linifanib as compared to the sorafenib arm, the predefined non-inferiority margin for overall survival (non-inferiority margin, 1.0491) was not exceeded.

### 4.4. Sorafenib plus Erlotinib

Erlotinib is an orally active, potent selective inhibitor of the EGFR/HER-1-related tyrosine kinase enzyme that shows a complementary effect to sorafenib in combined treatment, because of the lack of effect of sorafenib on the EGFR kinase activity. In anticipation of the additional effect of erlotinib, a phase III trial of sorafenib plus erlotinib *vs.* sorafenib plus placebo was conducted [46]. However, both groups of advanced HCC patients showed rather similar overall survivals and times to progression, and no additive effect of erlotinib could be demonstrated.

### 4.5. Vandetanib

Vandetanib is a small-molecule tyrosine kinase inhibitor that exerts inhibitory effect on the VEGFR and EGFR kinases, in contrast to sorafenib, which has no effect on the EGFR kinase activity. A randomized phase II trial of vandetanib 300 mg/day or vandetanib 100 mg/day *vs.* placebo was conducted to evaluate the tumor stabilization rate in unresectable HCC patients with no prior history of chemotherapy as the primary endpoint [47]. Vandetanib did not improve the tumor stabilization rates, although there was an insignificant trend towards improved progression-free survival and overall survival.

### 4.6. Nintedanib

Nintedanib is a triple angiokinase inhibitor of VEGF, FGF and PDGF signaling, with lower levels of activity against RET, Flt-3 and Src. Two randomized phase II trials of nintedanib *vs.* sorafenib in the first line setting were conducted in advanced HCC patients in Asian [48] and European countries [49], however, neither revealed any benefit of the drug on either the survival or the time to progression in the patients.

### 4.7. Dovitinib

Dovitinib inhibits FGFR as well as VEGFR and PDGFR. A phase II trial of dovitinib *vs.* sorafenib as first-line therapy in patients with advanced HCC revealed no significant benefit of the drug on either the survival or the time to progression as compared to sorafenib [50]. In addition, some adverse events, including diarrhea, decreased appetite, nausea and vomiting, fatigue, rash, and pyrexia occurred at significantly high frequencies (more than 30%) in the dovitinib arm.

### 4.8. Sorafenib plus Doxorubicin

In a randomized phase II trial of sorafenib plus doxorubicin *vs.* doxorubicin alone in patients with advanced HCC and Child-Pugh class A, treatment with sorafenib plus doxorubicin was associated with a greater median time to progression, overall survival, and progression-free survival as compared to doxorubicin monotherapy [51]. Considering this result of favorable overall survival, the possibility of synergism between sorafenib and doxorubicin was considered. Therefore, a phase III trial of sorafenib plus doxorubicin *vs.* sorafenib alone was carried out in patients with advanced HCC and Child-Pugh class A in Cancer and Leukemia Group B, however, the results were announced to be negative at the American Society of Clinical Oncology meeting 2015.

## 5. Systemic Chemotherapy: Second-Line Chemotherapy

A number of randomized trials of a variety of new agents, such as brivanib [52], everolimus [53], S-1 [54], axitinib [55], GC33 [56], tigatuzumab [57], *etc.*, *vs.* placebo have been conducted for advanced HCC patients refractory or intolerant to sorafenib. However, these clinical trials failed to demonstrate any clear survival benefit, and there was no established standard chemotherapeutic regimen for these HCC patients. Representative results of trials of the newer agents in the second-line setting are shown in Table 2.

### 5.1. Brivanib

A double-blind, randomized, placebo-controlled trial of brivanib was conducted in HCC patients who had already received treatment with sorafenib [52]. However, brivanib showed no significant beneficial effect on the overall survival, the primary endpoint, although it significantly delayed the time to progression.

### 5.2. Everolimus

The phosphatidylinositol 3-kinase/Akt/mammalian target of rapamycin (mTOR) pathway, a key regulator of cellular growth, proliferation, angiogenesis and survival, is a novel therapeutic target for HCC. Everolimus serves as an inhibitor of the mTOR pathway. In a phase III trial of everolimus *vs.* placebo in HCC patients with Child-Pugh class A liver function whose disease had progressed during or after sorafenib treatment or who were intolerant of sorafenib [53], everolimus showed no beneficial effect on either the overall survival or the time to progression. 

### 5.3. S-1

S-1 is an orally administered anticancer agent consisting of a mixture of tegafur and two modulators, gimeracil and oteracil, that was developed with the aim of intensifying the antitumor effect of 5-FU by increasing the serum concentration of the drug and mitigating its gastrointestinal toxicity. A placebo-controlled phase III trial of S-1 was conducted in Japan in patients with advanced HCC who were refractory to sorafenib [54]. However, no significant prolongation of the overall survival as compared to that in the placebo group was observed in sorafenib-refractory advanced HCC patients treated with S-1. On the other hand, a favorable effect on the progression-free survival was noted, and a subgroup analysis revealed a tendency towards improved overall survival in patients with stage III/IV and Child-Pugh class A.

### 5.4. Axitinib

Axitinib is a potent and selective VEGFR 1–3 inhibitor. To evaluate the efficacy and safety of axitinib, a global, randomized, placebo-controlled phase II trial was conducted [55]. The trial revealed no significant improvement of the overall survival in the treatment arm as compared to the placebo arm, although a significantly longer progression-free survival and higher disease control rate with acceptable toxicity were recognized in patients with advanced HCC.

### 5.5. GC33

GC33 is a humanized mAb directed against human glypican-3 (GPC3), which is highly expressed in the HCC tissue in >70% of cases; it exerts its antitumor effect through inducing antibody-dependent cytotoxicity (ADCC). A randomized phase II trial of GC33 *vs.* placebo was conducted to evaluate the efficacy of this mAb in patients of advanced HCC with a history of prior treatment [56]. However, no benefit was observed in the GC33 group as compared to the placebo group. 

### 5.6. Tigatuzumab

Tigatuzumab is a humanized monoclonal antibody that acts as a death receptor-5 agonist and exerts tumor necrosis factor-related apoptosis. A randomized phase II trial of tigatuzumab (6 mg/kg loading dose, 2 mg/kg/week maintenance dose) or tigatuzumab (6 mg/kg loading dose, 6 mg/kg/week maintenance dose) plus sorafenib *vs.* sorafenib alone as first-line treatment was conducted in patients with advanced HCC [57]; however, combined use of tigatuzumab with sorafenib had no effect of delaying the time to progression as compared to that in the sorafenib-alone arm.

In summary, sorafenib has come to be acknowlegded as the standard and first-line treatment agent for advanced HCC patients, because it has been demonstrated to significantly delay the time to progression and prolong the survival time in patients with advanced HCC in a phase III placebo-controlled study. After the advent of sorafenib, various newer agents have been evaluated in randomized controlled trials worldwide, however, none of the trials has yielded any significant positive or negative results and no newer agents that are superior to sorafenib in the first-line setting or to placebo in the second-line setting have emerged until date in this era of sorafenib.

## 6. Future: Post-Sorafenib Era

At present, various molecular-targeted agents, such as lenvatinib and resminostat for the first-line setting, or regorafenib and cabozantinib for the second-line setting, *etc.*, are under development worldwide for the treatment of advanced HCC patients (Table 3).

Recently, some molecular-targeted agents, such as ramucirumab and tivantinib, have been reported to show better efficacy in the biomarker-enriched population as compared to the whole population. Individualized cancer treatment using molecular-targeted agents based on the results of genome sequencing has begun to attract much interest in clinical practice. Also, some favorable outcomes have been reported of treatment with immune-oncology agents, such as anti-CTLA-4 antibody and PD-1/PD-L1 antibody (Table 3).

**Table 3:** Results of main clinical trials of promising agents in patients with advanced HCC: Post sorafenib-era.

Agents	*n*	RR (%)	DCR (%)	TTP/PFS (Median: Months)	Hazard Ratio (95% CI)	*p*-value	OS (Median: Months)	Hazard Ratio (95% CI)	*p*-value	Phase/Name of Trial	Authors	Reported Year	Reference No.
Lenvatinib	46	23.9	82.6	9.4	-	-	18.3	-	-	Phase II	Okita K	2012	[58]
Resminostat + Sorafenib	26	ND	ND	4.7	ND	ND	8.0	ND	ND	Phase II	Bitzer M	2012	[59]
Resminostat	19	ND	ND	2.2	-		4.1	-					
Regorafenib	36	3	72	4.3	-	-	13.8	-	-	Phase II	Bruix J	2013	[60]
Cabozantinib	41	5	83	4.4	-	-	15.1	-	-	Phase II	Verslype C	2012	[61]
Ramucirumab	283	7	56	2.8	0.63 (0.52–0.75)	<0.0001	9.2	0.87 (0.72–1.05)	0.14	Phase III	Zhu AX	2015	[62]
Placebo	282	<1%	46	2.1			7.6						
Ramucirumab (AFP ≥ 400)	119	ND	ND	2.7	ND	ND	7.8	0.67 (0.51–0.90)	0.006	Phase III	Zhu AX	2015	[62]
Placebo (AFP ≥ 400)	131	ND	ND	1.5	-		4.2	-					
Tivantinib (All patients)	71	1.4%	43%	1.6	0.64 (0.43−0.94) *	0.04	6.6	0.90 (0.57−1.40)	0.63	Phase II		2013	
Placebo (All patients)	36	0%	31%	1.4	-		6.2	-			Santro A	2013	[63]
Tivantinib (High expression of cMET)	22	ND	ND	2.7	0.43 (0.19−0.97)	0.03	7.2	0.38 (0.18−0.81)	0.01	Phase II			
Placebo (High expression of cMET)	15	ND	ND	1.4	-		3.8	-			Santro A	2013	[63]
Tremelimumab	20	17.6%	76.4%	6.48	-	-	8.2	-	-	Phase II	Sangro B	2013	[64]
Nivolumab	41	19%	67%	ND	-	-	62% †	-	-	Phase I/II	El-Khoueiry AB	2015	[65]

* 90% confidence interval; † 1 year survival; RR, response rate; DCR, disease control rate; TTP, time to progression; PFS, progression free survival; OS, overall survival; ND, no data; AFP, α-fetoprotein (ng/mL).

## 7. Development of Newer Agents for All Advanced HCC Patients without Patient Selection Based on Biomarkers

### 7.1. Lenvatinib

Lenvatinib is a tyrosine kinase inhibitor of VEGFR2, RET, *etc.*, and a phase II trial of the drug as first-line treatment or second-line treatment was conducted in 46 patients with advanced HCC [58]. Favorable treatment outcomes were reported, with a response rate of 23.9%, median time to progression of 9.4 months, and median survival time of 18.3 months. A global phase III trial comparing lenvatinib and sorafenib in the first-line setting is currently under way (NCT01761266), and the final results are expected to be reported in the near future, as patient enrolment for this study has already been completed.

### 7.2. Sorafenib plus Resminostat

Resminostat is an orally bioavailable inhibitor of histone deacetylases (HDACs); it inhibits phosphorylation of 4E-BP1 and p70S6k, causing disturbance of the Akt signaling pathway. A randomized phase II trial of resminostat plus sorafenib *vs.* resminostat has been conducted in advanced HCC patients with radiological progression under first-line treatment with sorafenib [59]. Use of resminostat in combination with sorafenib was associated with a more favorable progression-free survival and overall survival than use of resminostat alone. A randomized phase I/II study of resminostat plus sorafenib in patients with advanced HCC with no previous history of systemic chemotherapy is currently ongoing (NCT02400788).

### 7.3. Regorafenib

Regorafenib is a multikinase inhibitor that targets kinases involved in angiogenesis, such as VEGFR1–3 or TIE2, oncogenesis, such as c-kit or Ret, and the tumor microenvironment, such as PDGFR or FGFR. In 36 HCC patients in whom the disease had progressed under sorafenib treatment, this drug showed acceptable tolerability and evidence of antitumor activity (disease control rate, 72.2%; median time to progression, 4.3 months; median survival, 13.8 months) [60]. Therefore, a further phase III trial of regorafenib *vs.* placebo is under way in HCC patients showing disease progression after sorafenib treatment (NCT01774344).

### 7.4. Cabozantinib

Cabozantinib is an orally available small-molecule tyrosine kinase inhibitor that blocks phosphorylation of MET and VEGFR2. In a phase II randomized discontinuation trial, encouraging clinical activity of the drug has been reported in both the first- and second-line settings in HCC patients (disease control rate, 78%; median progression-free survival, 4.4 months; median survival, 15.1 months) [61]. Thus, further investigation in a phase III trial has been initiated in HCC patients showing disease progression after prior systemic treatment (NCT01908426).

## 8. Development of Newer Agents for Biomarker Selected HCC Patients

### 8.1. Ramucirumab

Ramucirumab is a human IgG1 monoclonal antibody that specifically binds with a high affinity to the extracellular domain of human VEGFR-2. Ramucirumab blocks the interaction of VEGFR-2 and its ligands and inhibits endothelial proliferation and migration. In a previous trial, the drug did not significantly improve survival as compared to placebo in the whole enrolled population [62]. However, in patients with baseline serum α-fetoprotein concentrations of 400 ng/mL or more, ramucirumab treatment was associated with prolongation of the progression-free survival and overall survival as compared to the findings in the placebo arm. Therefore, another phase III trial of ramucirumab *vs.* placebo is under way in patients with elevated baseline serum α-fetoprotein concentrations (≥400 ng/mL) after first-line therapy with sorafenib (REACH-2) (NCT02435433).

### 8.2. Tivantinib

Tivantinib (ARQ 197) is a selective, orally available, small-molecule MET inhibitor that preferentially inhibits growth of cells, and induces apoptosis in human tumor cell lines expressing MET. A placebo-controlled randomized phase II study carried out in the west demonstrated that tivantinib administered as a single agent delayed the time to progression in patients with advanced HCC as compared to placebo [63]. In addition, for patients with MET-high tumors, the time to progression and overall survival were longer in the patient group treated with tivantinib than in the placebo group, and the hazard ratio in the enriched population for c-MET expression (Hazard ratio, 0.43; 90% confidence interval 0.19–0.97) was smaller than that in the whole population (Hazard ratio, 0.64; 90% confidence interval 0.43–0.94). On the basis of the promising results of the subgroup analysis carried out by the MET status, a large, randomized, double-blind, phase III trial is being started to assess the effect of tivantinib on the overall survival in a selected population of HCC patients with MET-high tumors (NCT01755767).

## 9. Development of Individualized Cancer Treatments Using Molecular-Targeted Agents Based on the Results of Genome Sequencing

As therapeutic research has shifted focus from cytotoxic agents to molecular-targeted drugs, the approach of genome sequencing has often been applied to HCC patients to discover the underlying molecular mechanisms and to identify novel oncogenes and tumor suppressors. Recent cancer profiling studies have focused on next-generation sequencing (NGS) [66]. Individualized cancer treatments based on targeted DNA and RNA sequencing using NGS technology in formalin-fixed paraffin-embedded (FFPE) samples of HCC have recently been applied in patients with advanced HCC. Some investigational studies of the mutational profile in HCC patients identified an average of 30–40 mutations per tumor, among which six to eight possible drivers of common mutations were in the TERT promoter, TP53, CTNNB1, ARID1A, and AXIN1 [67]. TERT is a central driver gene and a promising molecular target in HCC, and targeting of the high-prevalence activation of the Wnt β catenin pathway in HCC cells should also offer new therapeutic opportunities. In a study of the clinical and molecular backgrounds of responders to sorafenib treatment who showed significant tumor shrinkage, FGF3/FGF4 amplification was observed in 3 of the 10 HCC samples from responders with evaluable DNA samples [68]. Thus, FGF3/FGF4 amplification is considered to be a possible mechanism involved in the response to sorafenib.

Umbrella studies, which allow patients to be assigned to specific treatments based on the mutation profiles of their tumors and personalizing the approach with a higher probability of success, are certainly a novel approach to drug development. Enrichment strategies can be used to avoid over-treatment and save valuable resources, by matching the right drug to the right subgroup of patients. The umbrella design has already been adopted in HCC treatment, as exemplified first by the Liver Cancer Center Heidelberg [69], and some clinical trials of molecular-targeted agents based on the results of genome sequencing, such as of a Wnt β catenin pathway inhibitor for patients with tumors carrying the CTNNB2 mutation, an FGFR4 inhibitor for those with tumors carrying FGF19 amplification, and a cMET inhibitor for patients with tumors showing MET amplification, are underway under the umbrella of biomarker profiling. In the United States, a study of individualized cancer treatments using molecular-targeted agents based on the results of genome sequencing, and the National Cancer institute-Molecular Analysis for Therapy Choice (NCI-MATCH) protocol for any type of cancer including HCC, is ongoing [70] (NCT02465060). In Japan, the Screening project for individualized medicine in Japan project (SCRUM-Japan), similar to the NCI-MATCH protocol, which is mainly used for gastrointestinal cancer (UMIN000016344) and lung cancer (UMIN000010234), is currently ongoing now. Thus, precision medicines are built on a centrally performed molecular portrait and molecularly selected cohorts with matched drugs, and individualized cancer treatments using molecular-targeted agents based on the results of genome sequencing are in progress throughout the world.

## 10. Development of Immune-Oncologic Agents for Advanced HCC Patients

Tumor immunotherapy is a promising, novel treatment strategy that may lead to improvements in both treatment-associated toxicities and outcomes. Among several immunotherapies, some immune checkpoint inhibitors, such as anti-cytotoxic T-lymphocyte-associated antigen 4 (CTLA-4) antibody [64] and anti-programmed death 1 (PD-1)/programmed death-ligand 1 (PD-L1) antibody [65], have recently been reported to provide promising outcomes.

### 10.1. Tremelimumab

The balance between co-stimulatory and co-inhibitory signals determines the degree of cytotoxic T-cell activation and intensity of the immune response. Immune checkpoint receptors are often upregulated in tumor tissues and promote tumor evasion from host immunosurveillance. CTLA-4, which is one of the immune checkpoint receptors, is expressed exclusively on activated T cells, Tregs, and naïve T cells. Tremelimumab is a monoclonal antibody that blocks CTLA-4, an inhibitory co-receptor that interferes with T cell activation and proliferation. A phase II trial has already been conducted in HCC patients with chronic hepatitis C viral infection [64]. The partial response rate and disease control rate were 17.6% and 76.4%, respectively, and the median time to progression was 6.48 months. Thus, a favorable treatment efficacy and good safety profile was obtained.

### 10.2. Nivolumab

Nivolumab is a fully human IgG4 PD-1 immune-checkpoint-inhibitor antibody; it disrupts the interaction between PD-1 and PD-L1/PD-L2 and may restore T-cell antitumor immunity directed against the tumor cells. A phase I/II trial of nivolumab across non-infected, HCV-infected, and HBV-infected patients has been performed in patients with advanced HCC [65]. It has a manageable toxicity profile in HCC patients, including those with HCV and HBV infection, and favorable responses were observed across all dose levels and all etiologic cohorts. In addition, two patients amazingly showed complete response following nivolumab treatment, and the overall survival rate at 12 months was 62%. Based on these promising results, a randomized phase III trial of nivolumab *vs.* sorafenib as first-line treatment for patients with advanced HCC will be planned (NCT02576509). Combination strategies with these immune-oncologic agents may increase the response rates to tumor immunotherapy. In fact, the tumor response and progression-free survival rates have been reported to be significantly greater in advanced melanoma patients treated with nivolumab plus ipilimumab [71], which is a monoclonal antibody that activate the immune system by targeting CTLA-4, than in those administered ipilimumab monotherapy. Therefore, studies are needed to determine which combinations would be the most effective. Furthermore, it is important to identify predictors of the response to these immuno-oncologic agents. PD-L1 expression has been reported to be predictive of benefit from nivolumab in patients with advanced non-small cell lung cancer [72], and mismatch repair-deficient tumors were highly responsive to checkpoint blockade with anti-PD-1 in patients with other solid tumors [73]. Thus, some immune-oncologic agents have been identified as potentially useful agents for systemic treatment of advanced HCC after sorafenib as well as for other solid tumors. Some clinical trials of a variety of anticancer agents, such as tremelimumab and PD-L1 antibody, MEDI4736 (NCT02519348) and nivolumab plus TGF-β inhibitor, galunisertib (NCT02423343), are being planned, and positive results are expected in the future.

## 11. Conclusions

Before the introduction of sorafenib, systemic chemotherapy was only of limited value in clinical practice, because some randomized controlled studies comparing promising regimens with single agents or no treatment failed to show any advantage in terms of the overall survival. Because two pivotal phase III trials demonstrated overt survival benefit of sorafenib in patients with advanced HCC, sorafenib has been acknowledged as a standard therapy for advanced HCC. The situation has changed greatly after the advent of sorafenib, but the efficacy of HCC treatments remains modest. A number of phase III trials of various molecular-targeted agents *vs.* sorafenib as a first-line treatment and of various molecular-targeted agents *vs.* placebo as second-line chemotherapy have been conducted, however, none of the agents examined so far has been demonstrated to provide any survival benefit over sorafenib or placebo. Various molecular-targeted agents in the biomarker-enriched population, individualized cancer treatments using molecular-targeted agents based on the results of genome sequencing, and immune-oncologic agents have begun to attract much interest in attempts at development of other effective chemotherapeutic agents following sorafenib. Thus, various novel systemic chemotherapeutic agents are currently under development, and further improvements in the treatment outcomes are expected. Hopefully, the international community will continue to witness meaningful progress in the treatment of patients with advanced HCC.
